# Validation of the Dysmorphic Concern Questionnaire (DCQ) in First‐Episode Schizophrenia

**DOI:** 10.1111/eip.70114

**Published:** 2025-11-17

**Authors:** Feten Fekih‐Romdhane, Nour Bouallègue, Amira Mohammed Ali, Carlos Laranjeira, Majda Cheour, Frederic Harb, Souheil Hallit

**Affiliations:** ^1^ The Tunisian Center of Early Intervention in Psychosis, Department of Psychiatry “Ibn Omrane” Razi Hospital Manouba Tunisia; ^2^ Faculty of Medicine of Tunis Tunis El Manar University Tunis Tunisia; ^3^ Department of Psychiatric Nursing and Mental Health, Faculty of Nursing Alexandria University Alexandria Egypt; ^4^ School of Health Sciences Campus 2, Polytechnic University of Leiria Leiria Portugal; ^5^ Centre for Innovative Care and Health Technology (ciTechCare) Polytechnic University of Leiria Leiria Portugal; ^6^ Comprehensive Health Research Centre (CHRC) University of Évora Évora Portugal; ^7^ Faculty of Medicine and Medical Sciences University of Balamand Kalhat Lebanon; ^8^ School of Medicine and Medical Sciences Holy Spirit University of Kaslik Jounieh Lebanon; ^9^ Applied Science Research Center Applied Science Private University Amman Jordan

**Keywords:** appearance, body dysmorphic disorder, Dysmorphic Concern Questionnaire, dysmorphic concerns, psychometric properties, schizophrenia

## Abstract

**Background:**

There is evidence that suggests that body dysmorphia represents a risk factor that may occur at the prodromal phase of schizophrenia, affecting ongoing developmental processes and conferring vulnerability to the disease. This study examined the psychometric properties of the Arabic Dysmorphic Concern Questionnaire (DCQ) among patients with a first‐episode schizophrenia in a Tunisian, Arabic‐speaking context.

**Method:**

A cross‐sectional study was conducted. Data collection was performed using a traditional paper‐and‐pencil approach by a single interviewer, who was one of the study's authors.

**Results:**

Our findings showed that the unidimensional factor structure of the DCQ holds up in patients with schizophrenia from a Tunisian culture and at an early stage of their disease, and shows excellent reliability (Cronbach's *α* of 0.91) in this specific population. Measurement invariance was supported in terms of three levels (configural, scalar and metric). Convergent validity of the DCQ was evidenced through significant positive correlations of the scale with abnormal bodily phenomena, muscle dysmorphia and body dissatisfaction. Besides, concurrent validity was demonstrated via significant positive correlations between the DCQ and four other measures: psychological distress, disordered eating, insight and psychotic symptoms severity.

**Conclusion:**

The DCQ showed good validity and reliability for measuring dysmorphic concerns in patients with schizophrenia from a Tunisian culture and at an early stage of their disease. The sound psychometric performance of the DCQ, its short administration time, as well as easy scoring and interpretability make it an excellent instrument for use in future clinical and research endeavours.

## 1 Introduction

Body dysmorphic disorder is a mental health condition characterised by excessive concern and preoccupation with perceived flaws or imperfections in one's physical appearance, which are often minor or not observable to others. Although it is generally regarded as a separate disorder by the DSM‐5‐TR (American Psychiatric Association 2022), researchers have suggested that some psychiatric disorders, such as schizophrenia, can present with non‐specific body dysmorphic concerns (BDC) (de Leon et al. 1989). Other authors have considered body dysmorphia as a variant of schizophrenia (Phillips et al. 2005). Recent reports suggest that body dysmorphia represents a risk factor that may occur at the prodromal phase of schizophrenia, affecting ongoing developmental processes and conferring vulnerability to the disease (Boland et al. 2021). There has also been evidence derived from case reports and case series to support that body dysmorphia could be an early presentation or a variant of schizophrenia (Abbassi et al. 2018; Durak et al. 2019; Lucchelli et al. 2006; Patel et al. 2004). A previous study demonstrated that people diagnosed with schizophrenia were significantly more likely to endorse appearance‐related concerns than non‐clinical controls (Rossell et al. 2020). A systematic review documented higher rates of body image concerns in individuals diagnosed with psychosis spectrum conditions compared to the general population (McAllister et al. 2024). Interestingly, research by Stanghellini et al. (Stanghellini et al. 2020) showed that abnormal bodily phenomena were more frequent and severe in schizophrenia than in bipolar disorder type I with psychotic features, and that these phenomena might be considered ‘stable trait‐characters’ and help in establishing more precise phenomenal boundaries between the two disorders. At the same time, more mixed findings have been reported in a systematic review, indicating that body image concerns were more present or not significantly different in people with psychosis spectrum conditions compared to the general population (McAllister et al. 2024).

Based on these recent findings, given the small body of evidence currently available on the topic, authors encouraged further investigations of the overlaps in key symptoms between BDC and schizophrenia (Rossell et al. 2020). Furthermore, nosological boundaries between body dysmorphic disorder and schizophrenia are debated, with the dichotomization of delusional thinking in body dysmorphia as either present or absent having been the object of controversies (Phillips 2004; Phillips et al. 1995). The DSM‐5 has partly addressed this concern by acknowledging the variation in the degree of insight regarding body dysmorphic disorder beliefs through an ‘insight specifier’ that can range from ‘good or fair’ to ‘absent/delusional’ (American Psychiatric Association 2022; Phillips et al. 2014). Other unresolved issues to be addressed include whether BDC that accompany schizophrenia influences its onset, therapeutic response, evolution and prognosis. There is therefore a crucial research need to develop a deeper understanding of what implications does the occurrence of BDC in people with schizophrenia has for the course of the disease. To achieve this, careful assessment of the psychometric properties of BDC measures in schizophrenia can be a first step towards accurate assessment of the construct in this specific population, ultimately answering the above questions and filling knowledge gaps.

### 1.1 Measurement Instruments of Body Dysmorphic Concerns in Schizophrenia

There exist a few empirical studies that attempted to identify and describe general features of body experiences in schizophrenia using various methods. Some of these studies focused on body image and functionality using self‐report measures (such as the Body Appreciation Scale, the Functionality Appreciation Scale (Mahfoud et al. 2023), the Body Image Questionnaire (Koide et al. 2002)), or using metric methods (such as the Image‐Marking Procedure, visual size estimation (Priebe and Röhricht 2001), the Figure Rating Scale (Loh et al. 2008)). Other studies adopted a qualitative approach (Stanghellini et al. 2019), or used a semi‐structured interview—the Abnormal Bodily Phenomena questionnaire (ABPq (Abnormi 2014)). The ABPq is designed to evaluate a range of anomalous body experiences specifically in patients with schizophrenia, and takes about 30–60 min. These measures either do not reflect the body dysmorphia construct, or can be costly, time‐consuming, burdensome and challenging to use as screening measures in resource‐constrained settings. In addition, their psychometric properties are unknown in individuals with schizophrenia. A systematic review and narrative synthesis of the literature which included 24 empirical studies on patients with psychotic disorders observed that 18 different measures were utilised to assess various body image aspects, among whom only three have been evaluated for psychometric properties (i.e., internal consistency) (McAllister et al. 2024).

One potential alternative that can be uniquely feasible, cost‐effective and promising for assessing BDC in people with schizophrenia is the Dysmorphic Concerns Questionnaire (DCQ (Mancuso et al. 2010; Oosthuizen et al. 1998)). The DCQ is a self‐report tool that was designed to screen for cognitive and behavioural components of body dysmorphia. It assesses both clinically significant and subthreshold concerns about physical appearance, without presuming the aetiology of the disorder (Jorgensen et al. 2001). The scale is made of seven items covering a single dimension with good reliability (Cronbach's alpha of 0.88) (Mancuso et al. 2010). The DCQ demonstrated adequate validity through significant correlations with depressed mood, social phobia and obsessive‐compulsive symptoms, professional and social impairment among 63 Australian in‐patients with severe mental illnesses (i.e., schizophrenia and related disorders, bipolar disorder, major depression, anxiety disorders) (Jorgensen et al. 2001; Oosthuizen et al. 1998). Due to its psychometric quality, brevity, simplicity and ease of administration, its use has been extended to community individuals and other clinical populations (e.g., patients with body dysmorphic disorder, dermatological outpatients) (Davies et al. 2022; Kapsali et al. 2020; Khanjani et al. 2019; Liao et al. 2010; Mancuso et al. 2010; Stangier et al. 2003). The DCQ has also been translated and validated into several languages, including German (Schieber et al. 2018), Greek (Kapsali et al. 2020), Spanish (Senín‐Calderón et al. 2017) and Persian (Khanjani et al. 2019). An Arabic‐language version of the DCQ has recently been made available (Fekih‐Romdhane et al. 2024). All the validations confirmed strong validity and reliability of the scale, including the Arabic version which showed excellent validity, internal consistency (McDonald's omega of 0.89), cross‐sex measurement invariance, and appropriate concurrent validity with measures of body appreciation, self‐esteem and disordered eating (Fekih‐Romdhane et al. 2024).

### 1.2 Rationale and Objectives

There are several reasons why this study is important and worth conducting. First, Kling et al. (2019) found in their systematic review that the suitability of body image measures differs across populations, cautioned against developing new measures, and called for extending the evidence available on the measurement properties of already existing measures. Second, the two validation studies conducted in psychiatric clinical settings involved a small proportion of patients with schizophrenia at the chronic stage of the disease. As body dysmorphia is suggested to occur since the early phases of the disease, it would be relevant to investigate the properties of the DCQ to screen body dysmorphic experiences in schizophrenia patients at their first episode. Third, BDC experiences may vary as a function of different cultural and linguistic backgrounds (Bohne et al. 2002). Therefore, the psychometric properties of the DCQ are yet to be specifically examined in schizophrenia patients from a different, non‐Western cultural context. Fourth, the Arabic version of the DCQ has been validated in a Middle Eastern Arab country (i.e., Lebanon), and its validation in other Arab regions and nations (such as North Africa) is still needed. This study examined the psychometric properties of the Arabic DCQ among patients with a first‐episode schizophrenia in a Tunisian context. It is expected that the scale will have good reliability, validity and measurement invariance across sex groups.

## 2 Methods

### 2.1 Design, Sample and Procedure

We carried out a cross‐sectional research study during the period between July 1st, 2024 and December 31st, 2024, in the Department of Psychiatry at Razi Hospital, Manouba, Tunisia. The study was conducted according to the STROBE (i.e., Strengthening the Reporting of Observational Studies in Epidemiology) guidelines (von Elm 2007). Eligibility criteria consisted of the following: (1) being an outpatient with schizophrenia defined according to the DSM‐5 criteria, with the diagnosis made by at least two trained psychiatrists (American Psychiatric Association 2013), (2) being aged 18 years and over, (3) being at the first episode of the disease, and having less than 3 months of treatment with antipsychotics (Compton et al. 2008), (4) being clinically stable at the time of the survey following previous studies' definition: ‘be symptomatically stable, as judged by the treating physician, be receiving a stable dose of an antipsychotic drug for at least 4 weeks before the survey and be in good general physical health’ (Fleischhacker et al. 2005) and (5) willing to participate. Participants who refused or failed to complete the questionnaire were excluded. Approval to undertake the research was approved by the ethics committee of Razi Hospital, Manouba, Tunisia. We obtained verbal informed consent from each patient before beginning the survey. The confidentiality of personal data and the voluntary nature of participation were ensured. Patients were informed that they have the right to withdraw their consent at any time without impact on their care or any penalty, and that they are free to decline or accept to take part in the research. Data were gathered by the same interviewer (i.e., one of the authors) using a traditional paper‐pen method. To ensure consistency, the interviewer administered sections requiring standardised delivery or clarification (e.g., sociodemographic and clinical items), while other sections consisted of validated self‐report questionnaires that patients completed independently under the interviewer's supervision.

### 2.2 Sample Size Calculation

We estimated a minimum sample of 21–140 participants based on the recommendation of 3–20 times per scale's variables (Mundfrom et al. 2005).

### 2.3 Measures

#### 2.3.1 The Dysmorphic Concern Questionnaire (DCQ)

The DCQ is a self‐administered tool that contains seven items evaluating concerns about one's physical appearance (Oosthuizen et al. 1998). Items are scored on a 4‐point Likert scale ranging between ‘not at all’ and ‘much more than most people’. The version translated, adapted and validated for the Arabic‐speaking context was used (Fekih‐Romdhane et al. 2024).

#### 2.3.2 The Anomalous Bodily Phenomena Questionnaire (ABPq)

The ABPq is a semi‐structured clinician‐rated interview that contains nine items split into five sections which reflect different atypical body phenomena experiences: Identity (experience of dysmorphophobia and transformation), Activity (recurrent pain‐like experiences and dysesthesia paroxysm), Coherence (experience of internal dynamisation), Vitality (devitalisation and morbid objectivisation) and Demarcation (experience of violation and externalisation) (Abnormi 2014). Items are scored on a 7‐point scale for their intensity, frequency, coping and impairment. Only intensity and frequency were rated in our sample, with Cronbach's alpha values of 0.78 and 0.77, respectively, in our sample.

#### 2.3.3 The Body Dissatisfaction Scale (BDS) of the Eating Disorder Inventory

The BDS is part of the Eating Disorder Inventory composed of nine items assessing body dissatisfaction (Garner et al. 1983). Items are rated on a four‐point scale from 0 (*never*/*rarely*/*sometimes*) to 3 (*always*). The version of the BDS validated in Arabic was used (Gerges et al. 2023), with a Cronbach's alpha value for total BDS score of 0.83 in our sample.

#### 2.3.4 The Muscle Dysmorphic Disorder Inventory (Ar‐MDDI)

The MDDI is a self‐report measure composed of 13 items assessing muscle dysmorphia through three dimensions (i.e., functional impairment, appearance intolerance, drive for size) (Hildebrandt et al. 2004). Each item is scored on a five‐point Likert‐type scale ranging from 0 (*never*) to 4 (*always*). The Arabic validated version of the MDDI was used (Fekih‐Romdhane, Merhy, et al. 2023; Fekih‐Romdhane, Maktouf, et al. 2023), which revealed a good internal consistency reliability in this sample (Cronbach's *α* of 0.78).

#### 2.3.5 The Assessment of Insight‐Expanded Version (SAI‐E)

The SAI‐E is a semi‐structured, clinician‐administered interview that was designed to assess levels of insight in individuals with psychotic disorders through 11 items reflecting the following dimensions: ability to rename psychotic phenomena as abnormal, awareness of having a mental illness and compliance with treatment (David 1990). The SAI‐E has been previously used in Tunisian patients with first‐episode schizophrenia (Fekih‐Romdhane, Merhy, et al. 2023; Fekih‐Romdhane, Maktouf, et al. 2023). Cronbach's alpha value for the total SAI‐E score was 0.90 in our sample.

#### 2.3.6 The Positive and Negative Syndrome Scale (PANSS)

The PANSS is an assessment tool for psychotic symptoms in patients with schizophrenia (Kay et al. 1987). It is a semi‐structured, clinician‐administered interview that contains 30 items scored on a seven‐point scale, and includes three dimensions (i.e., negative scale, positive scale and general psychopathological scale) (Hallit et al. 2017). In this study, Cronbach's *α* is of 0.78.

#### 2.3.7 The Eating Attitude Test‐7 (EAT‐7)

This is a self‐administered instrument composed of seven items assessing disordered eating symptoms (Fekih‐Romdhane et al. 2022). Each item is rated on a 5‐point scale as follows: *never to sometimes* = 0, *Often* = 1, *Usually* = 2 and *Always* = 3. The version translated and validated in Arabic of the EAT‐7 was utilised in the present sample (Fekih‐Romdhane et al. 2022), with a Cronbach's *α* of 0.85.

#### 2.3.8 The Depression, Anxiety, Stress Scales (DASS‐8)

This tool is a short form of the DASS‐21 (Lovibond and Lovibond 1995) that was validated in Arabic (Ali et al. 2022). It contains eight items split into three domains: stress (two items), depression (three items) and anxiety (three items). Response options range from ‘did not apply to me at all’ (0) to ‘Applied to me very much or most of the time’ (3). Greater scores indicate higher psychological distress in the three dimensions (Ali et al. 2024). Cronbach's *α* in this study was 0.90.

#### 2.3.9 Socio‐Demographic Information

All patients were asked to provide information on their sex, age, educational attainment, occupational status and marital status. Clinical variables gathered consisted of the duration of untreated psychosis (in months) and age at the onset of the disease.

### 2.4 Analytic Strategy

No missing data in the database. Confirmatory Factor Analysis (CFA) was carried out using SPSS AMOS v.30, employing the maximum likelihood estimation method to compute model parameters. To assess model fit, several indices were reported, including the standardised root mean square residual (SRMR ≤ 0.05), Comparative Fit Index (CFI ≥ 0.90), root mean square error of approximation (RMSEA ≤ 0.08) and Tucker‐Lewis Index (TLI ≥ 0.90) (Byrne 2013). Convergent validity was evaluated through the average variance extracted (AVE), with values ≥ 0.50 considered satisfactory (Malhotra 2020). Due to the violation of multivariate normality (Bollen‐Stine bootstrap *p* = 0.046), a non‐parametric bootstrapping method was applied.

Subsequently, a multi‐group CFA was conducted to test measurement invariance of the DCQ across sexes, examining scalar, metric and configural invariance levels (Chen 2007; Vadenberg and Lance 2000). Metric invariance indicates a consistent relationship between observed items and the latent construct, thus confirming that the factor loadings are equal across groups; configural invariance establishes that a latent construct's factor structure is the same across different groups, and scalar invariance allows for direct comparison of latent variable means by verifying that the item intercepts are equal across groups (Putnick and Bornstein 2016). ΔRMSEA ≤ 0.015 or ΔSRMR ≤ 0.010 and ΔCFI ≤ 0.010 provided evidence for invariance (Chen 2007). Between sex comparisons of DCQ scores were conducted using the Mann–Whitney test.

To evaluate internal consistency, both Cronbach's *α* and McDonald's *ω* were calculated, with coefficients exceeding 0.70 reflecting acceptable reliability (Malkewitz et al. 2023). Associations between DCQ scores and other constructs were analysed using Spearman's rank correlation.

## 3 Results

A total of 103 patients filled the survey, with a mean age of 27.46 years and 66.0% males. All other characteristics are summarised in Table 1. The mean DCQ‐7 total score was 6.99 ± 6.63, with 68.9% of patients endorsing at least one dysmorphic concern symptom.

**TABLE 1:** Patients' characteristics (*N* = 103).

	*N* (%)
Sex	
Male	68 (66.0%)
Female	35 (34.0%)
Educational attainment	
Primary	22 (21.4%)
Secondary	66 (64.1%)
University	15 (14.6%)
Occupational status	
Unemployed	62 (60.2%)
Student	10 (9.7%)
Day labourer	15 (14.6%)
Permanent employee	16 (15.5%)
Marital status	
Single	84 (81.6%)
Married	17 (16.5%)
Divorced	2 (1.9%)

	Mean ± SD
Age	27.46 ± 7.50
Age at onset of the disease (years)	24.91 ± 6.65
Duration of untreated psychosis (months)	30.94 ± 50.10

### 3.1 CFA

**FIGURE 1:**
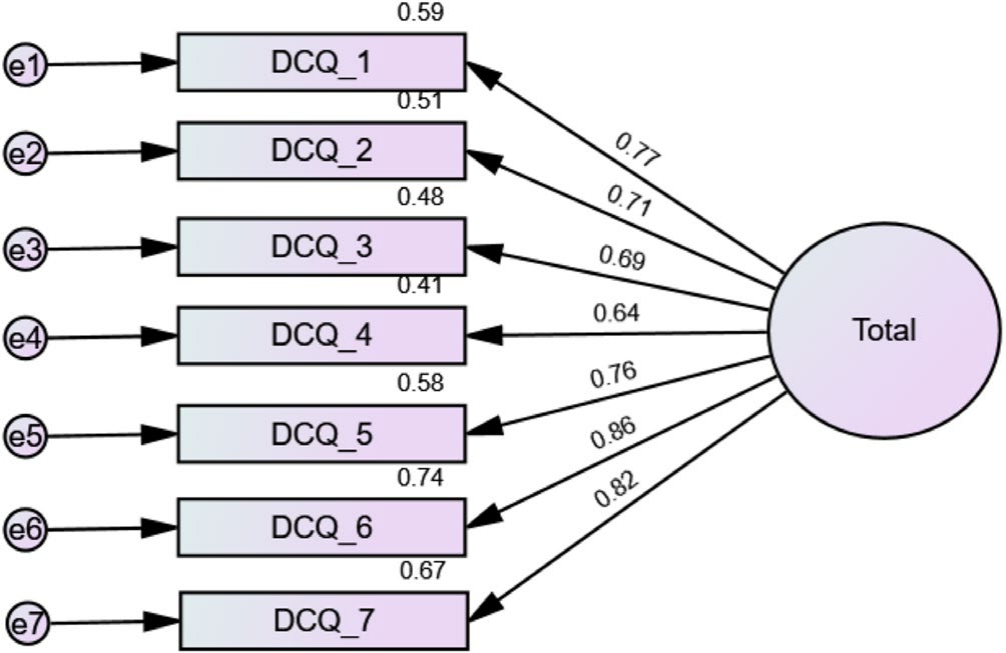
Standardised loading factors deriving from the confirmatory factor analysis of the one‐factor model of the Dysmorphic Concern Questionnaire (DCQ‐7).

The fit indices of the one‐factor model were acceptable (*χ* ^2^/df = 41.93/14 = 3.00; RMSEA = 0.140 (90% CI 0.092, 0.190), SRMR = 0.047, CFI = 0.936, TLI = 0.903). The standardised estimates of factor loadings were all adequate (Table 2). Internal reliability was adequate (*ω* = 0.91/*α* = 0.91). The AVE value was 0.57, supporting convergent validity. Standardised loading factors deriving from the CFA of the one‐factor model of the DCQ can be found in Figure 1.

**TABLE 2:** Frequency of occurrence of each dysmorphic concern and standardised loading factors deriving from the confirmatory factor analysis of the one‐factor model of the Dysmorphic Concern Questionnaire (DCQ‐7).

Item (Have you ever …)	Not at all	Same as most people	More than most people	Much more than most people	Mean ± SD	Loading factor
1. Been very concerned about some aspect of your appearance?	38 (36.9%)	8 (7.8%)	29 (28.2%)	28 (27.2%)	1.46 ± 1.24	0.77
2. Considered yourself misformed or misshapen in some way (e.g., nose/hair skin/sexual organs/overall body build)?	57 (55.3%)	13 (12.6%)	10 (9.7%)	23 (22.3%)	0.99 ± 1.25	0.71
3. Considered your body to be malfunctional in some way (e.g., excessive body odour, flatulence, sweating)?	57 (55.3%)	16 (15.5%)	18 (17.5%)	12 (11.7%)	0.85 ± 1.09	0.69
4. Consulted or felt you needed to consult a plastic surgeon/dermatologist/physician about these concerns?	75 (72.8%)	9 (8.7%)	10 (9.7%)	9 (8.7%)	0.54 ± 0.99	0.64
5. Been told by others/doctor that you are normal in spite of you strongly believing that something is wrong with your appearance or bodily functioning?	57 (55.3%)	13 (12.6%)	14 (13.6%)	19 (18.4%)	0.95 ± 1.20	0.76
6. Spent a lot of time worrying about a defect in your appearance/bodily functioning?	45 (43.7%)	9 (8.7%)	22 (21.4%)	27 (26.2%)	1.30 ± 1.27	0.86
7. Spent a lot of time covering up defects in your appearance/bodily functioning?	62 (60.2%)	9 (8.7%)	13 (12.6%)	19 (18.4%)	0.89 ± 1.21	0.82

### 3.2 Gender Invariance

The values of ΔCFI ≤ 0.010, ΔRMSEA ≤ 0.015 and ΔSRMR ≤ 0.010 supported the evidence of invariance of the DCQ‐7 scale between genders at the scalar, metric and configural levels (Table 3). No statistically significant difference was observed between males (Median (IQR) = [4 (0–10.75)] compared to females [4 (1–9)] in terms of dysmorphic concerns, *p* = 0.607, Cohen's *d* = 0.070).

**TABLE 3:** Measurement invariance of the Dysmorphic Concerns Questionnaire (DCQ‐7).

Model	CFI	RMSEA	SRMR	Model Comparison	ΔCFI	ΔRMSEA	ΔSRMR
Configural	0.814	0.182	0.056				
Metric	0.810	0.167	0.062	Configural vs. metric	0.004	0.015	0.006
Scalar	0.811	0.153	0.062	Metric vs. scalar	0.001	0.014	< 0.001

Abbreviations: CFI = comparative fit index; RMSEA = root mean square error of approximation; SRMR = standardised root mean square residual.

### 3.3 Discriminant and Concurrent Validity

Higher dysmorphic concerns scores were significantly associated with higher muscle dysmorphic disorder (rho = 0.40; *p* < 0.001; 95% CI 0.28–0.51), abnormal bodily phenomena frequency (rho = 0.73; *p* < 0.001; 95% CI 0.62–0.81) and intensity (rho = 0.72; *p* < 0.001; 95% CI 0.60–0.80) and body dissatisfaction (rho = 0.17; *p* = 0.011; 95% CI 0.04–0.31). In addition, greater dysmorphic concerns were positively correlated with disordered eating (rho = 0.21; *p* = 0.003; 95% CI 0.07–0.34), psychological distress (rho = 0.49; *p* < 0.001; 95% CI 0.37–0.59), PANSS total scores (rho = 0.22; *p* = 0.025; 95% CI 0.02–0.40) and insight levels (rho = 0.41; *p* < 0.001; 95% CI 0.23–0.56). The full correlation matrix can be found in Table S1. Item–total correlations of the DCQ can be found in Table S2. The Variance–Covariance Matrix of the DCQ is shown in Table S3.

The square root of the AVE value was 0.75, which was higher than the correlations between the factors, indicating discriminant validity.

## 4 Discussion

Findings showed that a unidimensional factor solution resulted in good fit in both sexes, and offered additional support to the excellent reliability of the DCQ. The measure is therefore easy to use, simple to interpret and appears to be valid based on expected patterns of correlations with various psychopathological variables. Furthermore, we found that 68.9% endorsed at least one dysmorphic concern symptom, which is in line with previous findings. A study showed that 17.9% of patients with first‐episode schizophrenia (*N* = 39) experienced at least one Dysmorphic‐like experience (Stanghellini et al. 2012). Another study found that abnormal bodily experiences, including dysmorphophobia, were found in 73.1% of ultra‐high risk (UHR) for psychosis subjects, whereas they were absent in all healthy controls (Madeira et al. 2016), suggesting that these bodily phenomena represent a relevant psychopathological feature of schizophrenia arising early in the course of the disease, even before the first psychotic episode (Klosterkötter and Schultze‐Lutter 2000; Röhricht and Priebe 2002; Schultze‐Lutter et al. 2007). Considering the mixed evidence available on higher rates of BDC in patients with psychotic disorders than the general population (McAllister et al. 2024), the present study has partially contributed to clarifying the contradictory findings of past research.

Despite the high prevalence rates of BDD symptoms and related impairment in functioning in mental and non‐mental health settings globally (McGrath et al. 2023), there still remains a lack of research on how the condition manifests across cultures and in the Middle East–North African region. Individuals from non‐white, non‐WEIRD (Western, Educated, Industrialised, Rich, Democratic) nations are largely underrepresented in the BDD literature (Hong et al. 2021; McGrath et al. 2023). This oversight is of concern since evidence suggests that culture may significantly influence body image perceptions (Hong et al. 2021). We could find no previous studies on BDD from Tunisia. This should prompt researchers to consider conducting future research on this topic of considerable public health importance.

Concerning the validity evidence based on the DCQ structure, CFA indicated that a one‐factor structure could represent the data observed for our sample of patients with a first‐episode schizophrenia. Our findings suggest that the unidimensional factor structure of the DCQ holds up in patients with schizophrenia from a Tunisian culture and at an early stage of their disease and shows excellent reliability (Cronbach's *α* of 0.91) in this specific population. Consistent with our findings, two previous studies carried out among Australian English‐speaking patients with schizophrenia at a chronic stage of the disease. The first one analysed data from 63 patients, among whom 33 had a diagnosis of schizophrenia or related disorders, and found that a one‐factor solution had a good fit to the data and a Cronbach's alpha of 0.88 (Oosthuizen et al. 1998). The second study included 65 patients with psychiatric conditions (9 of them had a primary diagnosis of schizophrenia) revealed that all items loaded into one factor with a Cronbach's *α* of 0.80 (Jorgensen et al. 2001). The DCQ was originally conceptualised as a unidimensional measure (Oosthuizen et al. 1998), and this single‐factor structure has been successfully replicated across various populations, cultures and nations (such as Germany (Schieber et al. 2018), Spain (Senín‐Calderón et al. 2017), Greece (Kapsali et al. 2020), United Kingdom (Monzani et al. 2012), United States (Rozzell et al. 2020), Iran (Khanjani et al. 2019)). Unidimensionality supports the general unity of the DCQ, meaning that the set of seven coherent items forming the measure assesses only one psychological construct and gives a single score of dysmorphic concerns. Hence, the DCQ total score is simple to interpret, meaningful, replicable, and may thus be useful in clinical and research settings. Furthermore, although the RMSEA value in the original model exceeded the conventional cutoff of 0.08, it is well established that RMSEA tends to be inflated when the degrees of freedom are low (df < 50) (Kenny et al. 2015; Kenny and McCoach 2003). Given that our model had a df of 14, RMSEA may not provide a reliable indication of model fit in this context. Therefore, greater emphasis should be placed on other indices, such as CFI and SRMR, which are considered more robust in these conditions (Lai and Green 2016). In the present study, both CFI and SRMR values fell within acceptable ranges, supporting an overall good model fit despite the elevated RMSEA.

Before testing DCQ mean differences across sex groups, measurement invariance was evaluated using multi‐group CFA and supported in terms of three levels (configural, scalar and metric). This suggests that factor loading is equivalent in male and female patients, and that the dysmorphic concerns construct has a similar meaning or structure for both sexes, ensuring that DCQ mean score comparisons can be meaningfully conducted and interpreted across sex groups. To our knowledge, this psychometric property has been previously established in only two studies, one among Lebanese community adults (Fekih‐Romdhane et al. 2024) and the other one among US sexual minority adults (Rozzell et al. 2020). The difference in DCQ total score between males and females was not significant in our sample. Our findings align with an earlier validation report of the DCQ among people with psychiatric conditions (including schizophrenia), which observed no significant correlation between DCQ total score and sex of patients (Oosthuizen et al. 1998). Studies on sex differences in BDC prevalence in samples of the general population have led to mixed and inconclusive findings, showing either no significant differences (Koran et al. 2008; Schneider et al. 2019) or a higher prevalence in females than males (16% vs. 11%, respectively) (McGrath et al. 2023). To date, the use of DCQ for sex comparisons is limited due to the very little information available about its invariance properties, and further investigations are needed to establish its utility for reliable comparison across sex. Although the absolute CFI values for the configural, metric and scalar models fell below the conventional cutoff of 0.90, it is important to note that absolute fit indices often appear deflated in small samples and complex models (Byrne 2013). Invariance testing relies more heavily on relative fit comparisons between nested models rather than on the absolute level of fit. In our analysis ΔCFI between successive models was ≤ 0.01 and ΔRMSEA was ≤ 0.15, both of which are widely accepted as indicators of invariance. Accordingly, despite modest absolute CFI values, the minimal changes in fit indices across levels of constraint supported configural, metric and scalar invariance. This approach is consistent with recommended practice, where stability of fit across nested models is prioritised over absolute thresholds when evaluating measurement invariance.

Convergent validity of the DCQ was evidenced through significant positive correlations between DCQ and measures of abnormal bodily phenomena, muscle dysmorphia and body dissatisfaction. Besides, concurrent validity was demonstrated via significant positive correlations between the DCQ and four other measures: psychological distress, disordered eating, insight and psychotic symptoms severity. Our results concur with those of previous research in which negative attitudes towards one's own physical appearance (i.e., body dissatisfaction) are a distinctive characteristic of body dysmorphia (Hrabosky et al. 2009), and disordered eating is a common consequence of overwhelming preoccupation with perceived physical defects (Mazzeo 1999; Mitchison et al. 2013). Dysmorphic concerns were found to be significantly related to the severity of psychotic symptoms in both clinical (Oosthuizen et al. 1998) and non‐clinical (Keating et al. 2016) populations. Research has similarly shown that individuals with body dysmorphic experiences tend to report greater anxiety and depression as well as a higher psychotic symptom endorsement than controls (Labuschagne et al. 2010). The current data along with those of the literature emphasise the clinical usefulness and applicability of DCQ to patients with early schizophrenia. Interestingly, DCQ scores were found to positively correlate with insight, which may appear counterintuitive at first glance. One possible explanation is that individuals who are able to report higher dysmorphic concerns in self‐report instruments may possess a greater level of metacognitive awareness of their preoccupations. Indeed, the act itself of completing a self‐report measure needs metacognitive capacity, which is closely related to clinical insight in schizophrenia (Lysaker et al. 2021). Future studies investigating the relationship between dysmorphic concerns and insight in schizophrenia should consider using clinician‐rated measures of BDD symptoms.

### 4.1 Limitations

To provide suggestions and guidance for future studies, a number of limitations to this study need to be mentioned. Convenience sampling used to recruit patients from a single clinical setting in Tunisia may limit the representativeness and generalizability of findings to the broader population of patients with first‐episode schizophrenia. Future validation studies are warranted to confirm the psychometric qualities of the DCQ for patients with different clinical characteristics (e.g., inpatients, UHR subjects) and from different cultural backgrounds. In addition, the consistency or stability of the measure over time and across raters still needs to be verified through analysis of test–retest reliability and interrater reliability. Information bias might have occurred as a result of inaccurate or misleading responses given by patients to the survey questions. Although the total sample of 103 patients was sufficient for conducting CFA, the relatively small female subgroup (*n* = 35) in the measurement invariance analyses may have limited statistical power and increased the likelihood of type II errors. Replication in larger and more balanced samples is therefore warranted to more reliably evaluate gender‐based measurement invariance and strengthen the robustness of our findings. Furthermore, the high percentage of males in our sample (66%) may limit the generalizability of the findings to the broader population of Arabic‐speaking patients with first‐episode schizophrenia because it may influence both the prevalence and expression of dysmorphic concerns. Consequently, the observed psychometric properties of the DCQ in this study may not fully reflect those of more diverse samples. Future studies are asked to take this limitation into consideration and confirm the robustness of the DCQ across different subgroups.

### 4.2 Practical Implications

Findings suggest that the DCQ is valid, reliable and suitable for assessing dysmorphic concerns in patients with schizophrenia. The sound psychometric performance of the DCQ in Tunisian culture and society, its short administration time, as well as easy scoring and interpretability make it an excellent instrument for use in clinical practice to evaluate symptoms and monitor their change during a therapeutic intervention. Of note, the DCQ is not intended to make a diagnosis; it is rather used as a screening tool to help in the diagnostic process. Additionally, proving the validity and reliability of the Arabic version of the DCQ in schizophrenia will hopefully open avenues for future research to decipher the clinical relevance of BDC on the course and treatment of the disease. Future studies should evaluate the test–retest reliability (which was not done in our study) and confirm the temporal stability of the DCQ in this population.

## 5 Conclusion

Our study contributes to immersion research and holds its significance since there are very limited or no studies that have looked at the psychometric properties of DCQ in an exclusively schizophrenia sample at the first episode. The measure showed good validity and reliability for measuring dysmorphic concerns, and can therefore be applied in routine practice. The reliance on DCQ will enable us to undertake future research on BDC in schizophrenia based upon a sound measure to help inform practice and policy decisions in this specific population.

## Ethics Statement

Each participant provided a voluntary oral informed assent before beginning the survey. The research protocol was approved by the ethics committee of the Razi Psychiatric Hospital, Manouba, Tunisia. The study was performed following the standards for medical research involving human subjects recommended by the Declaration of Helsinki for human research.

## Consent

The authors have nothing to report.

## Conflicts of Interest

The authors declare no conflicts of interest.

## Supporting information


**Table S1:** Correlation matrix.
**Table S2:** Item–total correlations of the Dysmorphic Concern Questionnaire.
**Table S3:** Variance–Covariance Matrix of the Dysmorphic Concerns Questionnaire.

## Data Availability

The data that support the findings of this study are available on request from the corresponding author. The data are not publicly available due to privacy or ethical restrictions.
