# Supplementary material for: Validation of the Dysmorphic Concern Questionnaire (DCQ) in First‐Episode Schizophrenia
Source: Early Interv Psychiatry. 2025 Nov 17;19(11):e70114. doi: 10.1111/eip.70114 (PMC12620770; doi:10.1111/eip.70114)
Supplement: Supplementary file 1 — Table S1: Correlation matrix. Table S2: Item–total correlations of the Dysmorphic Concern Questionnaire. Table S3: Variance–Covariance Matrix of the Dysmorphic Concerns Questionnaire. [file EIP-19-0-s001.docx]

**Supplementary Table 1.** Correlation matrix.

|  | Median | IQR | Mean | SD | 1 | 2 | 3 | 4 | 5 | 6 | 7 | 8 |
| --- | --- | --- | --- | --- | --- | --- | --- | --- | --- | --- | --- | --- |
| 1. Body dysmorphic concern | 4.00 | 13 | 5.61 | 5.71 | 1 |  |  |  |  |  |  |  |
| 2. Muscle dysmorphic disorder | 8.00 | 10 | 9.48 | 7.44 | 0.40*** | 1 |  |  |  |  |  |  |
| 3. Abnormal bodily phenomena - frequency | 18.00 | 13 | 18.63 | 9.11 | 0.73*** | 0.36*** | 1 |  |  |  |  |  |
| 4. Abnormal bodily phenomena - intensity | 18.00 | 14 | 18.84 | 9.34 | 0.72*** | 0.37*** | 0.97*** | 1 |  |  |  |  |
| 5. Body dissatisfaction | 15.00 | 5 | 14.88 | 5.25 | 0.17* | 0.02 | 0.32** | 0.35*** | 1 |  |  |  |
| 6. Disordered eating | 0.00 | 2 | 3.34 | 5.38 | 0.21** | 0.11 | 0.33*** | 0.31** | 0.28*** | 1 |  |  |
| 7. Psychological distress | 8.00 | 12 | 9.05 | 6.68 | 0.49*** | 0.39** | 0.51*** | 0.54*** | 0.16* | 0.05 | 1 |  |
| 8. PANSS total | 76.00 | 25 | 75.82 | 16.34 | 0.22* | 0.16 | 0.40*** | 0.36*** | 0.28** | 0.02 | 0.30** | 1 |
| 9. Insight | 14.00 | 15 | 13.16 | 8.62 | 0.41*** | 0.31** | 0.33*** | 0.36*** | 0.07 | 0.15 | 0.56*** | -0.18 |

*p < 0.05; **p < 0.01; ***p < 0.001. Numbers in the table correspond to Spearman correlation coefficients (rho).

Supplementary Table 2. Item–total correlations of the Dysmorphic Concern Questionnaire.

|  | 1 | 2 | 3 | 4 | 5 | 6 | 7 |
| --- | --- | --- | --- | --- | --- | --- | --- |
| 1. Total | 1 |  |  |  |  |  |  |
| 2. Item 1 | 0.85 | 1 |  |  |  |  |  |
| 3. Item 2 | 0.71 | 0.57 | 1 |  |  |  |  |
| 4. Item 3 | 0.74 | 0.57 | 0.51 | 1 |  |  |  |
| 5. Item 4 | 0.65 | 0.42 | 0.47 | 0.50 | 1 |  |  |
| 6. Item 5 | 0.79 | 0.60 | 0.51 | 0.56 | 0.57 | 1 |  |
| 7. Item 6 | 0.86 | 0.68 | 0.59 | 0.58 | 0.43 | 0.64 | 1 |
| 8. Item 7 | 0.78 | 0.55 | 0.49 | 0.55 | 0.57 | 0.59 | 0.72 |

***p < 0.001 for all correlations.

Supplementary Table 3. Variance–Covariance Matrix of the Dysmorphic Concerns Questionnaire.

|  | DCQ_1 | DCQ_2 | DCQ_3 | DCQ_4 | DCQ_5 | DCQ_6 | DCQ_7 |
| --- | --- | --- | --- | --- | --- | --- | --- |
| DCQ_1 | 1.148 | 0.677 | 0.555 | 0.412 | 0.666 | 0.833 | 0.641 |
| DCQ_2 | 0.677 | 1.072 | 0.520 | 0.461 | 0.570 | 0.702 | 0.570 |
| DCQ_3 | 0.555 | 0.520 | 0.915 | 0.434 | 0.570 | 0.595 | 0.528 |
| DCQ_4 | 0.412 | 0.461 | 0.434 | 0.797 | 0.536 | 0.446 | 0.559 |
| DCQ_5 | 0.666 | 0.570 | 0.570 | 0.536 | 1.134 | 0.757 | 0.666 |
| DCQ_6 | 0.833 | 0.702 | 0.595 | 0.446 | 0.757 | 1.254 | 0.880 |
| DCQ_7 | 0.641 | 0.570 | 0.528 | 0.559 | 0.666 | 0.880 | 1.075 |

Diagonal = item variances; Off-diagonal = covariances between items.
